# Saul-Wilson Syndrome Missense Allele Does Not Show Obvious Golgi Defects in a *C. elegans* Model

**DOI:** 10.17912/micropub.biology.000373

**Published:** 2021-03-04

**Authors:** Isabella Zafra, Benjamin Nebenfuehr, Andy Golden

**Affiliations:** 1 Lab of Biochemistry and Genetics, National Diabetes and Digestive and Kidney Diseases, NIH

## Abstract

Saul-Wilson Syndrome is an ultra-rare skeletal syndrome caused by a mutation in the COG4 gene resulting in a glycine-to-arginine substitution at amino acid position 516. The COG4 gene encodes one of 8 subunits of the conserved oligomeric Golgi complex. Using CRISPR-Cas9, our lab generated a *C. elegans *model for Saul-Wilson Syndrome by recreating the same glycine-to-arginine substitution in the worm ortholog *cogc-4*. Upon observation, the *cogc-4(av107) *worms did not display any obvious differences compared to wild-type worms. We used a variety of assays including stressing the worms using heat and Paraquat, as well as RNAi against the 7 other COG complex subunit genes in an attempt to uncover a phenotype. Our data suggest that this mutation in *cogc-4(av107)* worms does not lead to a detectable phenotype. Further studies should aim at more directly assessing Golgi function in this disease model.

**Figure 1:**
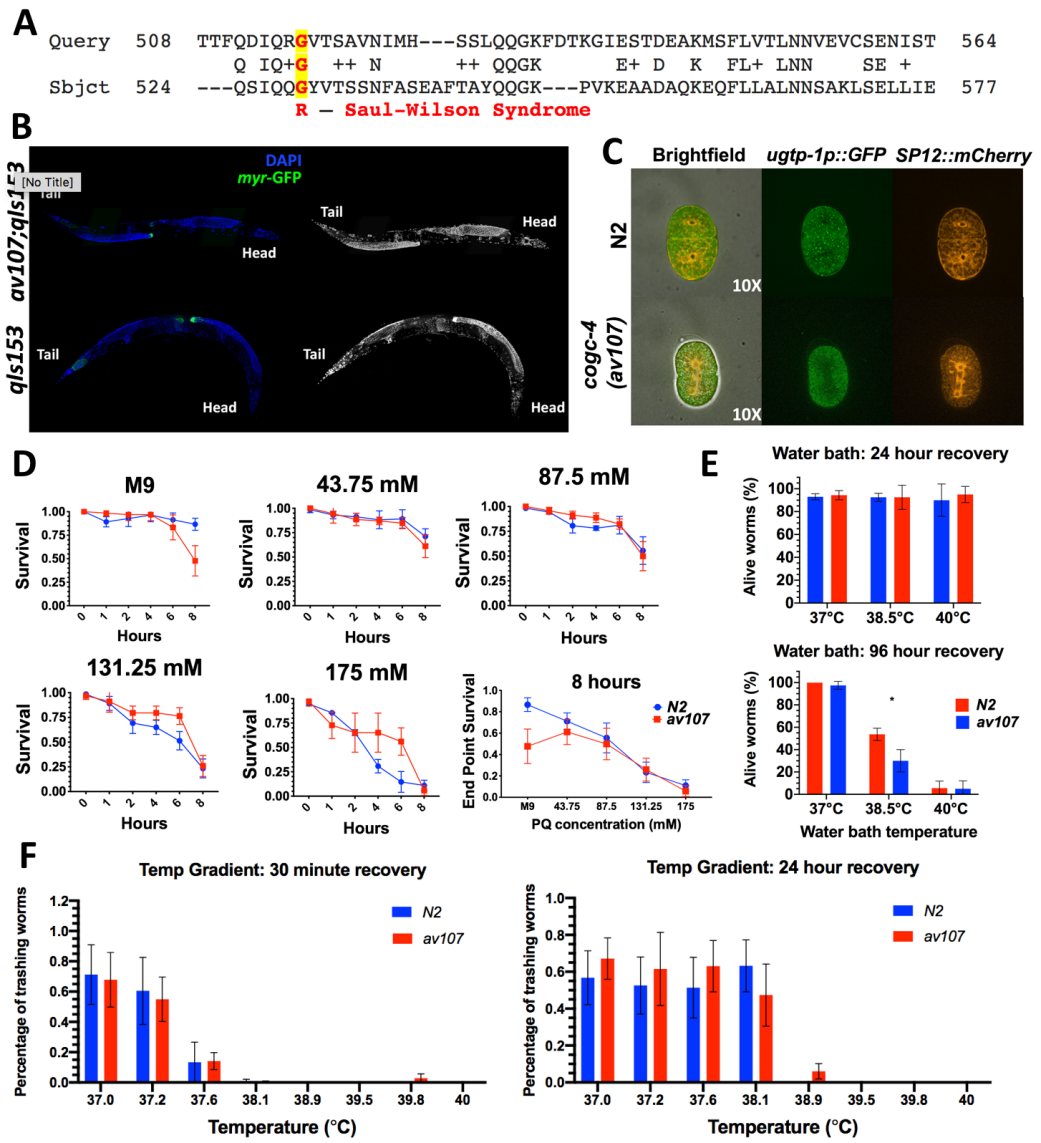
A. Patient mutation and alignment of region of interest in human COG4 and *C. elegans* COGC-4. B. No distal tip cell migration defects observed in adult animals. The distal tip cell marker used allows visualization of the plasma membrane of *lag-2* expressing cells, specifically of the distal tip cell. C. No co-localization of ER (SP12::GFP) and Golgi (*ugtp-1*::mChr) markers observed in 2-cell embryos. D. Paraquat Survival Assay. Time points represent hours after young L4 worms were placed in a 96-well plate containing various concentrations of Paraquat in M9. Each well was stirred for about 3 seconds with a wooden toothpick before worms were counted as either dead or alive. Worm counts were done at the beginning (immediately following the ‘0 hour’ counts) and after 8 hours. Worms were counted as “alive” if visibly thrashing, or if any type of movement was observed following “stirring” or directly after being poked with the wooden stick. Similarly, worms were counted as “dead” if no movement at all was observed for 5-10 seconds after stirring the well or poked directly. Sample size was 5-10 worms per well, 4 replicates per condition. Survival was compared at each Paraquat concentration using two-way ANOVA (genotype x timepoint) with Sidak multiple comparison’s test. There was no significant difference in survival between N2 and *cogc-4* worms. E. Heat shock: Water Bath. Percentage of N2 and *cogc-4(av107[G529R]*) L4 stage worms that were alive and visibly crawling 24 and 96 hours, respectively, after 3 hours at three different heat shock temperatures (37°C, 38.5°C and 40°C). Sample size was 30-50 worms per plate, 3 replicates per temperature. Visible thrashing after each heat shock was compared using two-way ANOVA (genotype x temperature) with Sidak multiple comparison’s test. There were significantly less N2 worms visibly thrashing following the 3 hour water bath at 38.5°C, only (*P = 0.0078). F. Heat Shock: Temperature Gradient. Across all eight temperatures assayed, both N2 and *cogc-4(av107[G529R]*) worms displayed similar levels of response immediately following heat shock (30 minute recovery) and 24 hours after the heat shock (24 hour recovery). Sample size was 5-10 worms per tube, 3 replicates per temperature. Visible thrashing was compared using two-way ANOVA (genotype x temperature) with Sidak multiple comparison’s test. There was no significant difference in survival between N2 and *cogc-4* worms.

## Description

Saul-Wilson Syndrome (SWS) is an ultra-rare, autosomal dominant skeletal dysplasia syndrome discovered in 1990; only 16 patients have been identified to date (Saul and Wilson 1990; Ferreira *et al.* 2018, OMIM#: 618150). The disease is characterized by short stature, various craniofacial abnormalities, shortened fingers and toes, and speech and physical developmental delay (Ferreira 2020). SWS is caused by a missense mutation in the *COG4* gene, resulting in a G516R residue change. Other pathogenic mutations have been observed in this gene and all are clustered at the C-terminal end of the protein (R724W, R729W, R729A, E764A). These are associated with Congenital Disorder of Glycosylation type 2j (CDGIIj). This is a recessive disease characterized by mild psychomotor delay, mild dysmorphic features, epilepsy, and defective sialylation (Reynders *et al.* 2009). Besides the mild developmental delay, this disease seems to share virtually no phenotypic similarity with SWS.

Human COG4 is one of 8 protein subunits that comprise a bi-lobed complex known as the Conserved Oligomeric Golgi Complex or COG complex (Smith and Lupashin 2008). This complex is involved in intra-Golgi trafficking and glycoprotein modification (Reynders *et al.* 2009). All *COG* genes, with the exception of *COG3*, have been implicated in recessive Congenital Disorders of Glycosylation (CDGs).

In assessing the clinical phenotype, Ferreira and others (2018) have reported data from a cohort of 14 SWS patients. These individuals did not have obvious glycosylation defects in serum proteins analyzed, although the secreted proteoglycan decorin appeared to be at a higher molecular weight than decorin secreted from control cells. This suggests some types of Golgi processing compartments are affected in SWS. Besides this observation, cellular trafficking in fibroblast cultures appeared normal and there was also no defect in COG subunit mRNA, protein expression, or localization. Immunofluorescence showed that nearly half of SWS patient fibroblasts had abnormal Golgi morphology characterized by collapsed stacks, while control fibroblasts had over 93% normal Golgi stacks. After treatment with Brefeldin A (BFA), a compound known to inhibit transport between the Golgi and ER, patient cells appeared to have a collapsed Golgi nearly twice as soon after treatment compared to control cells. This was evidenced by cis- and trans-Golgi markers becoming co-localized, and was termed “accelerated anterograde transport”– thus, attributing gain-of-function status to this G516R mutation in the mutant COG4 protein (Ferreira *et al.* 2018).

Two zebrafish models homozygous for frameshift mutations and both resulting in a truncated COG protein also revealed morphological Golgi defects, as well as inner ear deformities and decreased number of hair bundles in mechanosensory hair cells (Ferreira *et al.* 2018). In addition, biochemical findings included defects in proteoglycan secretion, as well as some types of collagen having decreased expression in certain tissues. These findings in the zebrafish models as well as from patient cultures have established a role for COG4 in inner ear development and skeletogenesis, which is, through some unknown mechanism, associated with defective Golgi structure. To this date, there has not been a model organism model studied in which the conserved Saul-Wilson allele has been mutated.

Given the moderate degree of amino acid conservation (29% amino identity, UniProt; 65.7% similarity, LALIGN) between human COG4 and the *C. elegans* ortholog COGC-4 (Fig. 1A), we decided to recreate the Saul-Wilson allele in the worm and assay for any Golgi-related phenotypes. On a macroscopic level, the worms did not display any morphological, reproductive, locomotive, or other behavioral defects. In addition, RNAi of each of the other 7 *COG* subunit genes individually did not reveal an obvious phenotype in these *cogc-4(av107)* worms as they were phenotypically indistinguishable from N2 worms. However, it should be noted that gene knockdown quantification was not verified with qRT-PCR, therefore there is the potential for incomplete knockdown.

It has been reported that RNAi of the individual COG complex lobe 1 subunit genes (*cogc-1, cogc-2, cogc-3 and cogc-4*, respectively) reveal gonad arm and distal tip cell (DTC) migration defects (Kubota *et al.* 2006). Therefore, we crossed in a DTC marker, but the *av107* worms did not display distal tip cell migration defects using the distal tip cell marker, despite the *cogc-4* RNAi DTC migration defect phenotype reported in Kubota *et al.* 2006 (Figure 1B). We also crossed in fluorescent Golgi and ER markers to monitor for evidence of Golgi collapse. Normal ER and Golgi morphology and no evidence of co-localization was observed in our *cogc-4(av107)* early embryos (Fig. 1C).

Given its role in Golgi trafficking, we then decided to perform a variety of stress assays—using heat and oxidative stress— in an attempt to uncover a phenotype (Fig. 1D-F). We reasoned that stressing worms could exacerbate a stronger and quantifiable phenotype. We used the methods described in Senchuk *et al.* (2017) as a reference in addition to troubleshooting to optimize the conditions such as Paraquat concentration and exposure time as well as optimal temperature ranges that worked best for our assays. There did not appear to be a difference in both survival after heat shock or recovery after heat shock for embryos or L4 stage worms between the two genotypes at any of the conditions tested. This is also true for survival in the presence of Paraquat (PQ) (Fig. 1D). It should be noted that all assays were performed using animals homozygous for the G529R mutation. We will continue to test these animals for phenotypes under a variety of other stresses and challenges.

## Methods

Generation of Saul-Wilson Allele using CRISPR: A single guide RNA (sgRNA) was designed that targeted the region surrounding position G529 in *cogc-4* (5’–ATGAGGTGACGTATCCCTGC– 3’). The DNA repair oligo (5’–GATACAGTCGTCCACGGGCATTTACGCCAGAGCATACAACAA**CGT**TACGTCACCTCATCCAATTTCGCATCTGAAGCATT– 3’) was designed to contain a mutated PAM site, 3 silent mutations, the desired G529R change and a restriction site for AclI used in genotyping for edits (underlined in the sequence shown). Missense mutations were identified using the following primers: forward 5‘- CTCAATCGTCGACGATGTGG; and reverse 5’- TTGATAGTGTCTGTGCCACC.

Heat Shock: Water Bath: A preliminary assay was performed using bleach-synchronized embryos being treated at 37°C or 38.5°C for 30 minutes or 180 minutes. Embryos were checked for viability and development into fertile adults 24 and 96 hours following the assay. However, at 37°C, 100% of N2 and *cogc-4(av107)* embryos hatched and developed into fertile adults after both the 30 minute and 180 minute exposure times. At 38.5°C, 100% of N2 and *cogc-4(av107)* embryos died after both 30 minute and 180 minute exposure times. We then decided to assay L4 stage worms in order to perhaps see more subtle phenotypes. Bleach-synchronized worms were allowed to reach L4 stage and were then incubated for 3 hours at 37°C, 38°C, or 40°C in a water bath. L4s were counted immediately after heat shock, and allowed 24 hours for recovery at 20°C, as well as assessed 96 hours after the heat shock treatment. Worms were assessed by eye for viability and movement by either poking them with a pick or by observing thrashing after a 1 ul drop of M9 was pipetted onto them on the agar plate.

Heat Shock: Temperature Gradient: Approximately ~1000 embryos were synchronized and placed onto multiple plates. Once the worms reached the L4 stage, worms were rinsed off of the plate(s) in a total of 2 ml of M9 and were added to two (2) separate Eppendorf tubes. 30 ul of worms in M9 were added into each of the tubes of an 8-tube PCR strip. Tubes were placed in the thermocycler and a heat gradient was run for 30 minutes that was programmed to the following eight temperatures: 37.0°C, 37.2°C, 37.6°C, 38.1°C, 38.9°C, 39.5°C, 39.8°C, and 40°C. Five replicates were performed for both genotypes (N2 and *av107*). After the 30 minutes, the contents of each tube were pipetted and aliquoted into a 96-well plate for assessment of survival. Worms were counted and assessed for thrashing or signs of viability 30 minutes after heat shock and 24 hours after the shock. They were gently poked with a platinum wire tip to check for movement.

Paraquat Survival Assay: *cogc-4(av107)* and N2 populations were bleach-synchronized, plated, and the embryos allowed to develop until the desired stage had been reached (L3-young L4s). Paraquat (PQ) solutions were prepared from a 200 mM stock, diluted with M9 to get final concentrations of 43.75 mM, 87.5 mM, 131.25 mM and 175 mM PQ solutions (accounting for the 5 ul of worms in M9 added to each well in later steps). 35 ul of each solution was added to the corresponding wells of a 96-well plate. Worms were washed off the plates using M9 and into an Eppendorf tube. M9 was either removed or added in order to make the final concentration of worms ~1 worm/ul. Worms were observed immediately after being added to the paraquat solution (time point 0), after 1 hour (T1), 2 hours (T2), and then every 2 hours until 8 hours had passed since being placed in the wells (T3-T5). At each time point, each well was stirred for about 3 seconds with a wooden toothpick before the number of moving worms per well was counted. Worms were counted as “alive” if visibly thrashing, or if any type of head movement was observed following “stirring” or directly after being gently poked with the wooden stick. Similarly, worms were counted as “dead” if no movement at all was observed after being stirred or poked directly. Total worms per well were counted at the beginning of the assay (immediately following the T0 viability count) and after the T5 counts.

Statistical analyses were performed for all Paraquat assay plots and temperature assays using GraphPad Prism 9.0 Software.

Microscopy: Images were captured by high-resolution confocal microscopy with Nikon 60 X 1.2 NA water objective with 1μm z-step size, and 60% intensity for the 488 nm and 561 nm lasers. Images were generated by custom Fiji Image J>Stacks>Z project (Linkert *et al.* 2010, Schindelin *et al.* 2012).

## Reagents

Strains: The Saul-Wilson allele strain we generated by CRISPR is AG529: *cogc-4(av107) G529R*. The DTC marker was crossed in from strain JK4475 (*qIs153* [*lag-2p*::MYR::GFP + *ttx-3p*::DsRed] V). The ER marker used was crossed in from strain OCF15: *pie-1p*::mCherry::sp12::*pie-1* 3’UTR + *unc-119(+)*. The Golgi marker used was crossed in from strain WH351: *pie-1*::GFP::*ugtp-1* + *unc-119(+)*. The three marker strains were obtained from the CGC.

Paraquat Assays: Paraquat dichloride hydrate was purchased from Millipore Sigma (36541-100MG).
