# Corrigendum: Saul-Wilson Syndrome Missense Allele Does Not Show Obvious Golgi Defects in a C. elegans Model

**DOI:** 10.17912/micropub.biology.000379

**Published:** 2021-03-24

**Figure 1.**
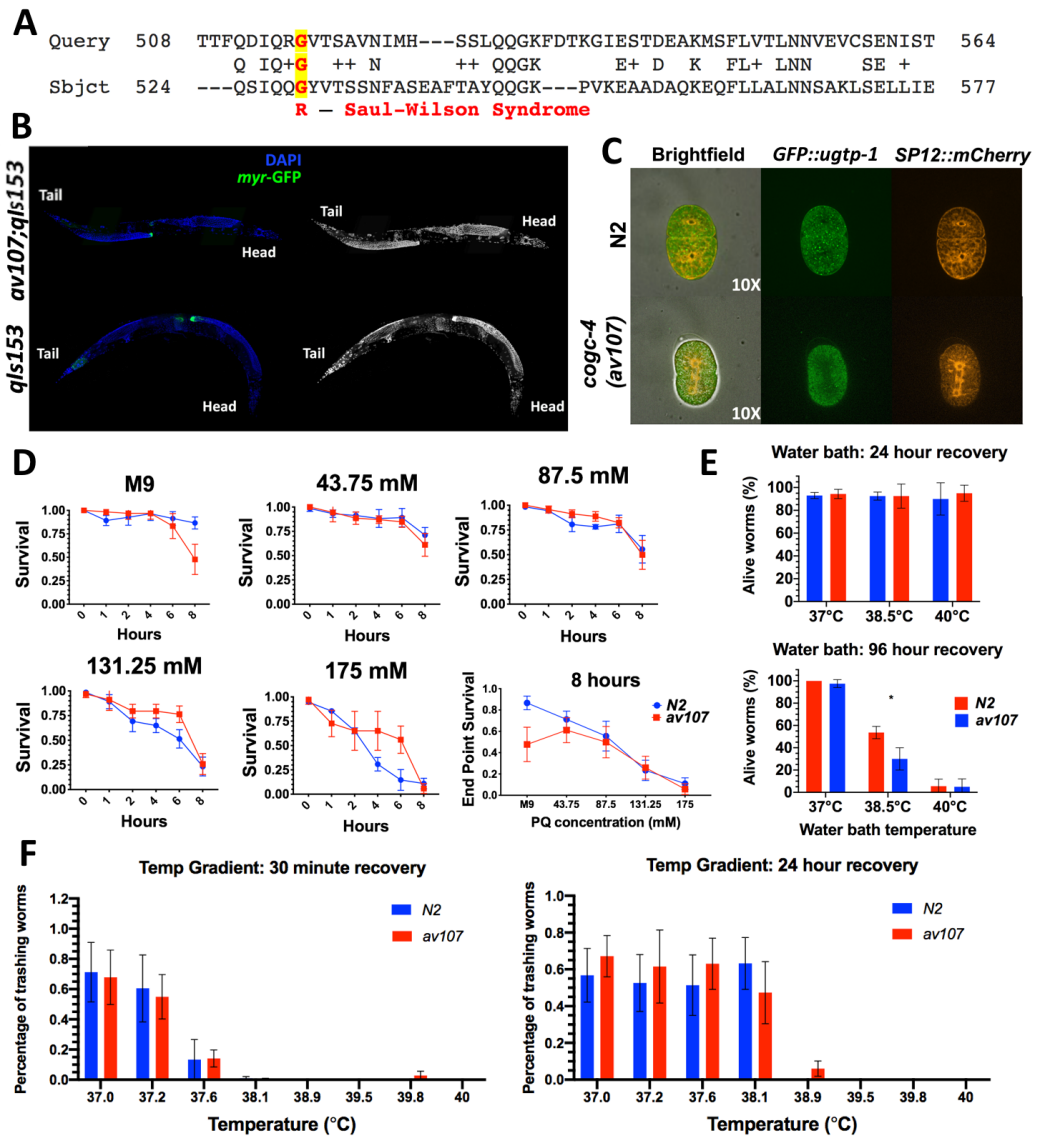


## Description

For Zafra, I; Nebenfuehr, B; Golden, A (2021). Saul-Wilson Syndrome Missense Allele Does Not Show Obvious Golgi Defects in a C. elegans Model. microPublication Biology. 10.17912/micropub.biology.000373.

The authors correct the following:

Figure: Panel C above the image of the embryo, the Golgi marker strain genotype “*ugtp-1p::GFP*”

is corrected to: “*GFP::ugtp-1*”

Figure legend: Where Panel C is described, it states, “No co-localization of ER (SP12::GFP) and Golgi (*ugtp-1::mChr*) markers observed in 2-cell embryos.”

This is now corrected to: “No co-localization of ER (SP12::mChr) and Golgi (*GFP::ugtp-1*) markers observed in 2-cell embryos.”.

Reagents: The Golgi marker strain genotype is written as “WH351: *pie-1::GFP::ugtp-1* + *unc119(+)*”

This is now corrected to: “WH351: *pie-1p::GFP::ugtp-1* + *unc119(+)”*.

